# Author response to: Comment on: Predictive value of pretreatment circulating inflammatory response markers in the neoadjuvant treatment of breast cancer: meta-analysis

**DOI:** 10.1093/bjs/znae190

**Published:** 2024-07-25

**Authors:** Gavin P Dowling, Gordon R Daly, Aisling Hegarty, Sandra Hembrecht, Aisling Bracken, Sinead Toomey, Bryan T Hennessy, Arnold D K Hill

**Affiliations:** Department of Surgery, Royal College of Surgeons in Ireland (RCSI) University of Medicine and Health Sciences, Dublin, Ireland; Medical Oncology Lab, Department of Molecular Medicine, Royal College of Surgeons in Ireland (RCSI) University of Medicine and Health Sciences, Dublin, Ireland; Department of Surgery, Beaumont Hospital, Dublin, Ireland; Department of Surgery, Royal College of Surgeons in Ireland (RCSI) University of Medicine and Health Sciences, Dublin, Ireland; Department of Surgery, Beaumont Hospital, Dublin, Ireland; Department of Surgery, Royal College of Surgeons in Ireland (RCSI) University of Medicine and Health Sciences, Dublin, Ireland; Department of Surgery, Beaumont Hospital, Dublin, Ireland; Department of Surgery, Royal College of Surgeons in Ireland (RCSI) University of Medicine and Health Sciences, Dublin, Ireland; Department of Surgery, Beaumont Hospital, Dublin, Ireland; Department of Surgery, Royal College of Surgeons in Ireland (RCSI) University of Medicine and Health Sciences, Dublin, Ireland; Medical Oncology Lab, Department of Molecular Medicine, Royal College of Surgeons in Ireland (RCSI) University of Medicine and Health Sciences, Dublin, Ireland; Medical Oncology Lab, Department of Molecular Medicine, Royal College of Surgeons in Ireland (RCSI) University of Medicine and Health Sciences, Dublin, Ireland; Department of Surgery, Royal College of Surgeons in Ireland (RCSI) University of Medicine and Health Sciences, Dublin, Ireland; Department of Surgery, Beaumont Hospital, Dublin, Ireland

*Dear Editor*

We would like to thank Chen *et al*.^1^ for the interest they have shown in our meta-analysis^2^.

We agree that publication bias is a limitation of our study; however, formal statistical assessments of this were not conducted due to the concerns discussed hereinafter. While publication bias assessment can add value to meta-analyses, Egger’s test, and its extensions, are not without limitations; in particular disproportionately high type I error rates^3^. Furthermore, Sterne *et al*.^4^ demonstrated that the power of Egger’s test was low for meta-analyses of ten or fewer trials, as was the case for the only analysis in our study that was found to exhibit publication bias (the analysis of the association between the white cell count and a pCR; only 3 studies were included in this analysis). Moreover, the significant heterogeneity of the studies included in our analysis, as highlighted by Chen *et al*.^1^, necessitates careful interpretation of publication bias assessments. In fact, Ioannidis and Trikalinos^5^ suggested that it may not be appropriate to use publication bias tests when significant heterogeneity is present (when the *I*^2^ statistic is greater than 50%). A random-effects model was applied in our study for analyses with significant heterogeneity. In keeping with the PRISMA guidelines, risk of reporting bias and quality assessment was conducted using the STROBE^6^ scores for each study included in our meta-analysis; these are available in the Supplementary material^2^. For our study, we felt this method of reporting bias was more suitable than Egger’s test for the aforementioned reasons.

We would like to thank Chen *et al*.^1^ for further highlighting the heterogeneity as a result of including various molecular subtypes, cut-off values, and patient cohorts. We agree that these are significant sources of heterogeneity and that inclusion of sufficient detail on these potential confounders in future studies would significantly strengthen our ability to derive definitive conclusions. Indeed, these limitations are all addressed at length in the Discussion section in paragraphs 7 and 8.

The inherent heterogeneity and in some cases the limited number of studies in our analyses seriously limits the value of adjusting calculations for publication bias in our study.
